# Changes of Peel Essential Oil Composition of Four Tunisian Citrus during Fruit Maturation

**DOI:** 10.1100/2012/528593

**Published:** 2012-05-01

**Authors:** Soumaya Bourgou, Fatma Zohra Rahali, Iness Ourghemmi, Moufida Saïdani Tounsi

**Affiliations:** Laboratoire des Substances Bioactives, Centre de Biotechnologie à la Technopole de Borj-Cédria Hammam Lif 2050, Tunisia

## Abstract

The present work investigates the effect of ripening stage on the chemical composition of essential oil extracted from peel of four citrus: bitter orange (*Citrus aurantium*), lemon (*Citrus limon*), orange maltaise (*Citrus sinensis*), and mandarin (*Citrus reticulate*) and on their antibacterial activity. Essential oils yields varied during ripening from 0.46 to 2.70%, where mandarin was found to be the richest. Forty volatile compounds were identified. Limonene (67.90–90.95%) and 1,8-cineole (tr-14.72%) were the most represented compounds in bitter orange oil while limonene (37.63–69.71%), **β**-pinene (0.63–31.49%), **γ**-terpinene (0.04–9.96%), and *p*-cymene (0.23–9.84%) were the highest ones in lemon. In the case of mandarin, the predominant compounds were limonene (51.81–69.00%), 1,8-cineole (0.01–26.43%), and **γ**-terpinene (2.53–14.06%). However, results showed that orange peel oil was dominated mainly by limonene (81.52–86.43%) during ripening. The results showed that ripening stage influenced significantly the antibacterial activity of the oils against *Staphylococcus aureus*, *Escherichia coli*, and *Pseudomonas aeruginosa*. This knowledge could help establish the optimum harvest date ensuring the maximum essential oil, limonene, as well as antibacterial compounds yields of citrus.

## 1. Introduction

The genus Citrus of the family Rutaceae includes several important fruits such as oranges, mandarins, limes, lemons, and grapefruits. Citrus fruits are one of the important horticultural crops, with worldwide agricultural production over 80 million tons per year [1]. Although, the fruits are mainly used for dessert, they have important economic value for their essential oils. Citrus essential oils are obtained as byproducts of the citrus processing and are the most widely used essential oils in the world. In fact, among the great variety of essential oils, citrus fruit essential oils and their major components have gained acceptance in the food industry since they have been generally recognized as safe, and many foods tolerate their presence [2].

Citrus essential oils have wide uses. Primarily, they are used as aroma flavour in many food products, including alcoholic and nonalcoholic beverages, marmalades, gelatins, sweets, soft drinks, ice creams, dairy products, candies, and cakes [3, 4]. In pharmaceutical industries they are employed as flavoring agents to mask unpleasant tastes of drugs. Additionally, in perfumery and cosmetic, the low volatile essential oil components play an important role as head notes (e.g., in Eaux-de-Cologne and soap perfumes) [3]. Recently, other applications make use of the major compound of the oil extracted from citrus peels, limonene, as green solvent for Soxhlet extraction [5] due to its ability to solubilize fats [6].

The chemical composition of the essential oil from citrus peels has been studied and reviewed [7–12]. However, there have been few research projects focusing on volatiles during citrus fruit ripening. Dugo et al. [13] investigated the seasonal variation of the chemical composition of the essential oil extracted from the whole fruit of two cultivars of Sicilian mandarin (*Citrus. deliciosa *Tenore. cv Avana and Tardivo di Ciaculli) and reported a decrease of limonene level at the beginning and the end of the season. According to Vekiari et al. [14] harvesting time is a critical parameter influencing significantly the chemical compositions of the Cretan lemon peel and leaf oil. Likewise, Droby et al. [9] analysed the composition of peel essential oil of various citrus cultivars including sweet orange, clementine, and grapefruit at different stages of maturity and found that limonene was the predominant compound through ripening.

Although the chemical composition of peel essential oil extracted from various Tunisian citrus varieties has been studied by Hosni et al. [15], data regarding the effect of ripening on the oil chemical composition as well as the effect of ripening stage on the antibacterial activity of the citrus oils have not been reported. Therefore, the objectives of this study were to evaluate the volatile profile during the maturation of four citrus fruits, in order to understand the significance of those compounds in the ripening process of this fruits and to determine the optimal accumulation period of desirable compounds and to evaluate the antibacterial activity variation during ripening. Information on the effects of ripening on oil composition and bioactivities is crucial to optimize harvesting protocols.

## 2. Materials and Methods

### 2.1. Materials

Fruits of Citrus of four species: bitter orange (*Citrus aurantium*) cultivar Larange, lemon (*Citrus limon*) cultivar Beldi, orange maltaise (*Citrus sinensis*) cultivar Lsen asfour, mandarin (*Citrus reticulate) *cultivar Elarbi, were evaluated in this study. Samples were collected at three harvesting periods: stage 1 (green colour, immature), stage 2 (yellow colour; semi-mature), and stage 3 (orange colour: mature), in 2009, from Menzel Bouzelfa in the North East of Tunisia (latitude 36°42′13′.17′′; longitude 10°29′46.93′′). The fruits peels including flavedo (epicarp) and albedo (mesocarp) layers were peeled off carefully and discarded.

#### 2.1.1. Essential Oil Isolation

The fresh peels (100 g) were submitted to hydrodistillation for 120 min using a Clevenger-type apparatus. This time was fixed after a kinetic survey during 30, 60, 90, 120, 150, 180, and 210 min. The oils obtained were dried over anhydrous sodium sulphate and stored at −20°C in darkness until analysed.

### 2.2. GC and GC-MS Analysis

Essential oils were analyzed by gas chromatography (GC) using a Hewlett-Packard 6890 apparatus (Agilent Technologies, Palo Alto, CA, USA) equipped with a flame ionization detector (FID) and an electronic pressure control (EPC) injector. A HP-Innowax capillary column (polyethylene glycol: 30 m × 0.25 mm i.d., 0.25 *μ*m film thickness; Agilent Technologies, Hewlett-Packard, CA, USA) was used; the flow of the carrier gas (N_2_) was 1.6 mL/min and the split ratio 60 : 1. Analyses were performed using the following temperature program: oven temps isotherm at 35°C for 10 min, from 35 to 205°C at the rate of 3°C/min, and isotherm at 205°C over 10 min. Injector and detector temperature were held, respectively, at 250 and 300°C.

### 2.3. Gas Chromatography-Mass Spectrometry

GC-MS analyses of essential oil volatile components were carried out on a gas chromatograph HP 5890 (II) coupled to a HP 5972 mass spectrometer (Agilent Technologies, Palo Alto, CA, USA) with electron impact ionization (70 eV). A HP-5MS capillary column (30 m × 0.25 mm, 0.25 *μ*m film thickness; Agilent Technologies, Hewlett-Packard, CA, USA) was used. The column temperature was programmed to rise from 50°C to 240°C at a rate of 5°C/min. The carrier gas was helium with a flow rate of 1.2 mL/min; split ratio was 60 : 1. Scan time and mass range were 1 s and 40–300 m/z, respectively.

### 2.4. Compounds Identification

Identification of essential oil volatile compounds was based on the calculation of their retention indices (RI) relative to (C_8_–C_22_) *n*-alkanes with those of authentic compounds available in our laboratory. Further identification was made by matching their recorded mass spectra with those stored in the Wiley/NBS mass spectral library of the GC-MS data systems and other published mass spectra [16].

### 2.5. Screening of Antibacterial Activity

Antibacterial activity was analyzed by the disc diffusion method [17] against three human pathogenic bacteria including Gram-positive *Staphylococcus aureus* (ATCC 25923), and Gram-negative bacteria *Escherichia coli *(ATCC 35218) and *Pseudomonas aeruginosa* (ATCC 27853). All bacteria were grown on Mueller Hinton plate at 30°C for 18–24 h previous inoculation onto the nutrient agar. A loop of bacteria from the agar slant stock was cultivated in nutrient broth overnight and spread with a sterile cotton swap onto Petri dishes containing 10 mL of API suspension medium and adjusted to the 0.5 McFarland turbidity standards with a Densimat (BioMerieux). Sterile filter paper disks (6 mm in diameter) impregnated with 10 *μ*L of essential oil were placed on the cultured plates. After 1-2 h at 4°C, the treated Petri dishes were incubated at 25 or 37°C for 18–24 h. The antimicrobial activity was evaluated by measuring the diameter of the growth inhibition zone around the discs. Each experiment was carried out in triplicate, and the mean diameter of the inhibition zone was recorded.

### 2.6. Statistical Analyses

All data are reported as means ± standard deviation of three samples. Statistical analysis was performed with STATISTICA. Differences were tested for significance by using the ANOVA procedure, using a significance level of *P* < 0.05.

## 3. Results and Discussion

### 3.1. Yields of Essential Oils

The essential oils yields of four citrus peels during fruit maturation are shown in Table 1. Species and harvest time had significant effect on essential oil yield. Independently to ripening stage, mandarin exhibited the highest yield (2.70%) followed by lemon (1.30%) and orange (0.74%) while bitter orange showed the smallest value of 0.46%.

On the other hand, essential oil yields varied during ripening to reach maximum values during the middle stage of maturity (stage 2) for mandarin and orange while the highest lemon yield was found at the beginning of fruit maturation and decreased after that. Bitter orange showed different pattern behaviour evolution from other species since the yield doubled during ripening from 0.23 at stage 1 to 0.46% at stage 3. Vekiari et al. [14] reported a seasonal variation of the yield of lemon peel essential oil extracted from Zambetakis variety cultivated in the island of Crete with the highest value reached at the middle of the season.

Our results concerning the mature stage are in accordance with those of Hosni et al. [15] who demonstrated that Tunisian mandarin peel was the richest on essential oil compared to orange and bitter orange. However, these authors reported higher values (varying from 1.24 to 4.62%). Such differences could be due to the effect of extraction procedure and environmental conditions. In fact, these authors used dried and ground material from citrus cultivated in Mograne region which is known to belong to the semiarid region, while in our experiment we used a fresh material collected from Menzel Bouzelfa which belongs to the humid region. Extractions parameters are known to greatly influence the essential oil yield [18, 19]; moreover, water supply during ripening was reported to influence considerably the essential oil content with an enhancement of the yield under moderate water shortage conditions [20, 21].

On the other hand, the yields obtained in our study were higher than those reported in the literature; Ahmad et al. [22] reported yields varying from 0.30 to 1.21% for the four citrus varieties from Pakistan. Moreover, lower yields were reported for the mandarins from France (yield ranging from 0.05% to 0.60%) by Lota et al. [8] and the mandarin from Colombia (yield of 0.79%) by Blanco Tirado et al. [23].

### 3.2. Essential Oil Composition

Analysis of citrus peel essential oils composition showed 39 identified compounds presenting fluctuations during ripening (Table 2). 

#### 3.2.1. Bitter Orange

Analysis of the essential oil indicated that it is made essentially from monoterpenes hydrocarbons which constitute the main class during ripening varying from 71.21 to 94.61% and reaching a maximum at full maturity (Table 2). Oxygenated monoterpenes were the second class. This later was present with appreciable levels at the first and the middle stages of maturity (7.75 and 22.51%); however, it was markedly reduced at maturity (2.11%). Sesquiterpenes were weakly represented during the ripening.

Analysis of the volatile composition showed the predominance of limonene which level varied from 67.90 to 90.95% during ripening, with the highest value reached at the maturity stage (Table 3). The relevance of limonene in mature bitter orange peel is in accordance with previous reports [15]. The essential oil was also characterized by appreciable levels of camphor (0.17–6.37%), cis-linalool oxide (tr-3.40%), *α*-terpinene (0.91–1.66%), and octanol (0.02–1.59%). Moreover, the results showed that particularly 1,8-cineole reached an important level of (14.7%) at the semimature stage.

Despite the dominance of limonene, the low-abundant compounds are known to contribute actively to citrus aroma. Thus, camphor, which has green dry leave note [24], could mainly influence the aroma in the first stage of ripening while 1,8-cineole, characterised by a fresh and cool aroma [25], could participate actively to the citrus aroma at the middle stage. However, several minor compounds including *α*-terpinene (lemon aroma) [26], *α*-terpineol (green and floral-like aroma) [27], and carvacrol may influence the aroma at the mature stage.

Interestingly, when limonene showed the lowest level at the middle stage (67.90%), several minor compounds including 1,8-cineole, terpinolene, sabinene hydrate, and linalool oxide reached their highest content. These later compounds are synthesised from a common precursor: *α*-terpinyl cation [28]. Thus, at middle stage the biosynthesis of limonene is lowered in favour to other cyclic monoterpenes mainly 1,8 cineole. Biosynthesis of 1,8-cineole is thought to proceed from the a-terpinyl cation via an *α*-terpineol intermediate which undergoes internal additional cyclization of the alcoholic oxygen [29].

#### 3.2.2. Lemon

As for bitter orange, essential oil composition of lemon was dominated by monoterpenes hydrocarbons followed by oxygenated monoterpenes (Table 2). These classes represented 98.83, 87.15, and 90.43% for monoterpenes hydrocarbons and 3.92, 8.72, and 5.61% for oxygenated monoterpenes during first, middle, and mature stages, respectively.

Immature fruit presented a limonene chemotype since it constituted the predominant compound with a percentage of 68.08% (Table 3). However, at semimature stage, limonene level decreased (37.63%) while *β*-pinene level (31.49%) increased. Thus, the essential oil chemotype becomes “limonene/*β*-pinene”. At mature stage, limonene was found to be the most abundant (69.71%).

Lota et al. [30] analyzed the volatile composition of peel oil of 43 taxa of lemon cultivated in France and reported different chemotypes including limonene and limonene/*β*-pinene/*γ*–terpinene. Besides, Flamini et al. [31] analysed the volatiles emitted by the pericarp of unripe and ripe Italian lemon fruit and found that limonene level enhanced from 65.30 at immature stage to 68.30% at mature stage. These authors concluded also that the emitted volatiles are originated from glandular structures which give an essential oil similar to fruit emissions.

On the other hand, other representative compounds in lemon oil were *γ*-terpinene and* p*-cymene (Table 3) which showed opposite behavior during ripening; in fact, *p*-cymene level decreased from 9.84% at immature stage to 0.23% at maturity while *γ*-terpinene level augmented to reach 9.96% at maturity. This is in accordance with their biosynthetic pathway, in which *γ*-terpinene is a precursor of *p*-cymene [32]. Further, the Germacrene D and valencene were the most abandon sesquiterpenes (Table 3). Minor compounds including valencene have stand out in citrus as important flavour and aroma compounds [33].

#### 3.2.3. Orange Maltaise

Differently to bitter orange and lemon, the peel essential oil of orange fruit extracted at three stages of maturity was mainly dominated by limonene which presented a level of 86.43, 81.52, and 85.35% at immature, semimature, and mature stages, respectively (Table 3). The rest of compounds were weakly represented with levels lower than 1% except for the monoterpenes *β*-pinene, camphor and bornyl acetate. These compounds reached their maximum at semimature stage with levels of 1.80, 4.81, and 4.21%, respectively. The increase of camphor and *β*-pinene levels suggests an activation of the related terpene synthases which catalyze their formation from the critical intermediates pinyl and bornyl cations, respectively [29]. Concerning sesquiterpenes, *α*-humulene (0.16–0.34%) followed by germacrene-D (tr-0.12%) were found to be the most represented compounds.

Our results are in accordance with that of Droby et al. [9] who analysed the composition of peel essential oil of sweet orange fruit at different stages of maturity and found that limonene was the predominant compound with percentage varying from 72.41 to 94.77%. However, these authors reported a decrease of limonene level during the course of fruit maturity especially at the end of the ripening. Such difference could be attributed to both interactions between genetic (biotic) and environmental (abiotic) factors since in their study, these authors considered “Valencia” and not maltaise cultivar.

On the other hand, concerning the mature stage, Hosni et al. [15] reported a higher limonene level (96.00%) in the peel essential oil extracted from Tunisian maltaise orange than that obtained in our study. These authors found that *β*-pinene was also a marked compound (1.82%). However, camphor was not detected in their samples.

#### 3.2.4. Mandarin

Analysis of peel oil composition of mandarin during ripening indicated that immature stage was characterized by the predominance of monoterpenes hydrocarbons where limonene (65.37%) followed by *γ*-terpinene (12.44%) were the main compounds. Moreover, borneol (1.22%) and caryophyllene oxide (1.18%) were the most represented oxygenated monoterpenes and sesquiterpenes, receptively (Tables 2 and 3).

Limonene and *γ*-terpinene levels decreased at semimature stage to 51.81 and 2.53%, respectively. This decrease was accompanied with a concomitant increase of 1,8-cineole (26.43%) and E-*β*-ocimene (7.93%) levels. The enhancement of the biosynthesis of these compounds suggests an activation of the related synthases at semimature stage. The cyclic monoterpenes including limonene, *γ*-terpinene, and *α*-terpineol are produced from a common intermediate: terpeiyl cation. *α*-terpineol undergoes after that cyclisation to lead 1,8-cineole. On the other hand, the enhancement of the acyclic terpene E-*β*-ocimene level suggest the activation of E-*β*-ocimene synthase which derives its conversion from linalyl cation [29]. Shimada et al. [34] reported that the expression of 1,8-cineole and E-*β*-ocimene genes and their related products in Satsuma mandarin was high in flower and then decreased toward mandarin fruit development. At maturity, 1,8-cineole and E-*β*-ocimene levels decreased. The essential oil was found to be dominated by limonene (69.00%) followed by *γ*-terpinene (14.06%). If considering only the mature stage, similar composition has been described by Lota et al. [7] who reported limonene (52.20–96.20%) and *γ*-terpinene (tr-36.70%) as the main compounds of French mandarins peel oils.

### 3.3. Antibacterial Activity

The antibacterial activity of citrus essential oils was tested against human pathogenic bacteria. The results presented in Table 4 showed great differences in the activity between citrus species and during ripening stages. The oils were effective against Gram (+) and Gram (−) bacteria, with a major activity against *S. aureus* and *E. coli*. Bitter orange, lemon, and orange were effective against *P. aeruginosa* only at maturity while mandarin oil remained inactive against this strain. In accordance with our finding, Espina et al. [12] reported no activity of the peel oil extracted from mature mandarin fruit against *P. aeruginosa*. On the other hand, lemon and mandarin essential oils extracted from immature fruit exhibited the highest antibacterial activity against *E. coli *which was comparable of positive control activity. In the case of *S. aureus*, the oils were mostly active at mature stage with mandarin oil showing the highest antibacterial activity.

The antimicrobial activity of essential oils is believed to be associated with phytochemical components especially monoterpenes [35] which diffuse into and damage cell membrane structures. However, the differences on the oils activity found in our experiment between ripening stages may be related to the modifications of the oils composition during fruit maturation. For the four citrus species, the variations of the activity were not correlated to that of the major compound level: limonene. This is in agreement with literature data where limonene is a weak antibacterial compound [36]. Moreover, the inhibitory activity of an essential oil is known to result from a complex interaction between its different constituents, which may produce additive, synergistic, or antagonistic effects, even for those present at low concentrations. Thus, at immature stage, antibacterial compounds including camphor [37] and *α*-thujone [38] in bitter orange, *α*-terpineol [39] and nerol in lemon [40], and borneol [37] and caryophyllene oxide [41] in mandarin may be involved in the found activities of the corresponding oils.

At semimature stage, the higher antibacterial capacity of orange oil compared to the citrus oils could be linked to the presence of camphor at appreciable level (4.81%). Moreover, borneol could be involved in the found activity since this monoterpene alcohol was reported to exhibit moderate antibacterial activity against *S. aureu*s and *E. coli *but to remain inactive against *P. aeruginosa *[42]. Our results demonstrated also an important increase of 1,8 cineole in bitter orange and mandarin oils. Literature data dealing with the activity of 1,8 cineole are contrasting; Randrianarivelo et al. [43] reported that this ether is effective against Gram(+) and Gram(−) bacteria including *E. coli* and *S. Aureus*; however, Hussain et al. [44] found that 1,8 cineole remained inactive against *P. aeruginosa* and *E. coli*. The low activity of the bitter orange and mandarin oils at the semimature stage in spite of a high level of 1,8 cineole suggests that this compound is inactive.

The activity of the four oils extracted from mature fruits could mainly be due to the presence of the phenolic compound carvacrol. This later is a well-known antibacterial compound acting at low concentration [38]. Moreover, mandarin was distinguished from other citrus species by the presence of farnesol which was reported to be highly effective against *S. aureus* [45] while the activity of orange oil could be ascribed to the enhancement of humulene percentage, which was reported to be moderately active [46].

## 4. Conclusions

This study revealed that the ripening stage affects significantly the yield and the composition of the examined Tunisian citrus. Immature stage offered the maximum yield for lemon while semimature fruit was the best ripening stage for mandarin and orange maltaise. In the case of bitter orange, maturity was the best stage. The oils chemical compositions and the antibacterial activity changed during ripening, and the maximum levels of the most abundant volatile compounds identified were dependent on maturity stage. For the four citrus, the highest limonene level was reached already at immature stage which suggests that at least in the case of lemon and for economic purposes, fruits could be harvested at immaturity in order to obtain essential oil with high yield and limonene content.

## Figures and Tables

**Table 1:** Yields (%) of peels essential oils from four cultivars of citrus at different ripening stages.

Ripening stage	Bitter orange	Lemon	Orange maltaise	Mandarin
Stage 1	0.23^Bb^	1.30^Aa^	0.13^Cb^	0.22^Cb^
Stage 2	0.12^Cd^	0.48^Bc^	0.74^Ab^	2.70^Aa^
Stage 3	0.46^Ac^	0.62^Bbc^	0.52^Bc^	1.13^Ba^

Means of three replicates. (Values with different superscripts are significantly different at *P* < 0.05). Caps superscripts (comparison between stages). Small superscripts (comparison between citrus species).

**Table 2:** Variations of levels (%) of chemical classes of essential oils obtained from four cultivars of citrus at different ripening stages.

Chemical classes	Ripening stage	Bitter orange	Lemon	Orange maltaise	Mandarin
Monoterpenes hydrcarbons	Stage 1	83.35^Bab^	89.83^Aa^	89.72^Aa^	84.73^Aab^
	Stage 2	71.21^Cb^	87.15^ABa^	87.12^ABa^	66.45^Bc^
	Stage 3	94.61^Aa^	90.43^Ab^	90.15^Ab^	89.57^Ab^
Oxygenated monoterpenes	Stage 1	7.75^Ba^	3.92^Cb^	1.50^Bc^	2.10^Bb^
	Stage 2	22.51^Aa^	8.72^Ab^	7.15^Ab^	28.68^Aa^
	Stage 3	2.11^Cc^	5.61^Ba^	1.82^Bd^	3.54^Bb^
Sesquiterpenes hydrocarbon	Stage 1	0.35^Aa^	0.06^Bb^	0.26^Aa^	0.01^Bb^
	Stage 2	0.44^Ab^	1.28^Aa^	0.28^Ab^	0.08^Bc^
	Stage 3	0.21^Abc^	1.39^Aa^	0.35^Ab^	0.34^Ab^
Oxygenated sesquiterpenes	Stage 1	0.42^Ab^	0.01^Ac^	0.01^Ac^	1.36^Ba^
	Stage 2	0.16^Bb^	0.01^Ac^	0.01^Ac^	0.27^Ca^
	Stage 3	0.09^Bb^	0.01^Ab^	0.01^Ab^	3.29^Aa^

Means of three replicates. (Values with different superscripts are significantly different at *P* < 0.05). Caps superscripts (comparison between stages). Small superscripts (comparison between citrus species).

**Table 3:** Variations of chemical composition (%) of essential oils obtained from four cultivars of citrus at different ripening stages.

Volatile compounds^A^	RI^a^	RI^b^	Ripening stage	Bitter orange	Lemon	Orange maltaise	Mandarin
			Stage 1	0.05^Aab^	0.16^Aa^	tr	0.09^Bab^
Tricyclen	924	1014	Stage 2	0.75^Aa^	0.021^Ab^	0.09^Ab^	0.14^Ab^
			Stage 3	0.0^Ab^	0.02^Ab^	tr	0.15^Aa^
			Stage 1	0.14^Ba^	0.38^Ab^	0.37^Ab^	1.31^Aa^
*α*-thujene	928	1035	Stage 2	0.27^ABa^	Tr	tr	0.12^Bb^
			Stage 3	0.44^Aa^	0.34^Aa^	0.20^Aa^	0.39^Ba^
			Stage 1	0.13^Bc^	1.29^Aa^	0.41^Ab^	0.03^Cd^
*α*-pinene	939	1032	Stage 2	0.03^Cb^	5.9^Aa^	0.44^Ab^	0.7^Bb^
			Stage 3	0.36^Ab^	1.14^Aa^	0.7^Aab^	1.25^Aa^
			Stage 1	0.24^Aab^	0.13^Bb^	0.07^Ab^	0.39^Aa^
Camphene	954	1076	Stage 2	tr	0.9^Aa^	1.22^Aa^	tr
			Stage 3	0.02^ABa^	0.03^Ba^	0.06^Aa^	0.09^Ba^
			Stage 1	tr	6.48^ Aa^	tr	1.31^Aa^
Sabinene	975	1132	Stage 2	tr	3.8^ Aa^	tr	0.0^Ba^
			Stage 3	0.20^Ab^	5.82^ Aa^	0.36^Ab^	0.18^Bb^
			Stage 1	0.11^Bd^	1.06^ Ab^	1.54^Ab^	0.43^Bc^
**β**-pinene	980	1118	Stage 2	0.07^Ba^	31.49^ Aa^	1.80^Aa^	0.06^Ba^
			Stage 3	0.38^Ab^	0.63^ Aa^	0.97^Ab^	0.75^Ab^
			Stage 1	0.09^Ab^	1.54^Aa^	tr	1.59^Aa^
Myrcene	991	1174	Stage 2	0.48^Aa^	Tr	tr	tr
			Stage 3	0.07^Aa^	0.99^Ba^	0.71^Aa^	0.98^Aa^
			Stage 1	tr	Tr	tr	tr
Δ-3-Carene	1011	1159	Stage 2	tr	Tr	tr	tr
			Stage 3	0.01^Aa^	Tr	0.05^Aa^	0.03^Aa^
			Stage 1	1.23^Aa^	0.24^Bb^	tr	0.30^Bb^
*α*-Terpinene	1018	1188	Stage 2	0.91^Aa^	Tr	tr	1.52^Aa^
			Stage 3	1.66^Aa^	1.05^Aa^	0.93^Aa^	0.73^Ba^
			Stage 1	tr	9.84^ Aa^	0.25^Ab^	0.63^Aa^
*p*-Cymene	1026	1280	Stage 2	0.09^Aab^	2.21^ Ba^	0.27^Aab^	0.68^Aa^
			Stage 3	0.08^Ac^	0.23^ Bb^	0.21^Abc^	0.7^Aa^
			Stage 1	80.54^Aa^	68.08^ Ab^	86.43^ Aa^	65.37^ Ab^
Limonene	1030	1203	Stage 2	67.90^ ABab^	37.63^ Bb^	81.52^ Aa^	51.81^ Aab^
			Stage 3	90.95^ Aa^	69.71^ Ab^	85.35^ Aa^	69.00^ Ab^
			Stage 1	tr	0.51^ABa^	tr	0.01^Bb^
1.8-Cineole	1033	1193	Stage 2	14.72^ Aab^	0.82^ Ab^	tr	26.43^ Aa^
			Stage 3	0.30^ Aa^	tr	tr	0.18^Bb^
			Stage 1	0.5^Ab^	0.14^Bc^	tr	0.77^Ab^
*E-*β**-Ocimene	1050	1266	Stage 2	0.09^Bab^	tr	0.09^Aa^	**7.93** ^ Aa^
			Stage 3	0.02^Bb^	0.5^Aab^	0.03^Ab^	1.05^Aa^
			Stage 1	0.14^ Ab^	0.04^ Bb^	tr	12.44^ Aa^
*γ*-terpinene	1058	1255	Stage 2	0.21^ Aa^	5.12^ ABa^	0.34^Aa^	2.53^ Ba^
			Stage 3	0.31^ Ac^	9.96^ Ab^	0.43^Ac^	14.06^ Aa^
			Stage 1	tr	0.024^Bb^	0.37^Aa^	0.04^Aa^
cis-Sabinene hydrate	1062	1550	Stage 2	0.18^Aa^	0.05^Aa^	0.26^Aa^	0.24^Aa^
			Stage 3	0.09^Bab^	tr	0.14^Aa^	0.04^Abc^
			Stage 1	0.05^Aa^	tr	tr	0.03^Ba^
Octanol	1070	1546	Stage 2	1.59^Aa^	tr	0.07^Aa^	1.41^Aa^
			Stage 3	0.02^Aab^	tr	tr	0.05^Ba^
			Stage 1	tr	tr	tr	tr
cis-Linalool oxide	1074	1450	Stage 2	3.40^Aa^	0.49^Ab^	0.82^Aab^	0.36^Ab^
			Stage 3	0.09^Bb^	0.0^Bd^	0.03^Ac^	0.14^Ba^
			Stage 1	0.16^Ab^	0.42^Aa^	0.25^Ab^	0.024^Cc^
Terpinolene	1092	1290	Stage 2	0.22^Aa^	tr	1.07^Aa^	0.7^Aa^
			Stage 3	0.02^Ab^	tr	tr	0.17^Ba^
			Stage 1	0.03^Ab^	0.0^Cc^	0.12^Aa^	tr
Linalool	1098	1553	Stage 2	tr	1.59^Aa^	0.54^Ab^	0.09^Ab^
			Stage 3	0.1^Aa^	0.65^Ba^	0.13^Aa^	0.67^Aa^
			Stage 1	0.12^Aa^	tr	0.21^Aa^	tr
Nonanal	1102	1400	Stage 2	tr	tr	0.03^Ba^	0.07^Aa^
			Stage 3	0.18^Aa^	tr	0.01^Bb^	0.05^Ab^
			Stage 1	0.35^Aa^	tr	0.06^Ab^	0.05^Ab^
*α*-Thujone	1115	1413	Stage 2	tr	tr	tr	0.03^Aa^
			Stage 3	0.09^Aab^	tr	tr	0.17^Aa^
			Stage 1	6.37^ Aa^	tr	tr	tr
Camphor	1144	1550	Stage 2	1.43^ Aa^	0.32^ABa^	4.81^Aa^	0.039^Aa^
			Stage 3	0.17^ Ab^	0.58^Aa^	0.17^Ab^	0.35^Aab^
			Stage 1	0.13^Ab^	0.33^Bb^	0.14^Ab^	1.22^Aa^
Borneol	1165	1719	Stage 2	0.16^Aa^	0.65^Ba^	0.7^Aa^	0.04^Ba^
			Stage 3	0.08^Ab^	2.54^Aa^	0.3^Ab^	0.04^Bb^
			Stage 1	0.05^Aa^	tr	tr	tr
Terpinene-4-ol	1178	1611	Stage 2	0.54^Aa^	1.28^Aa^	0.041^Ba^	0.026^Aa^
			Stage 3	0.01^Ab^	tr	0.26^Aa^	0.01^Ab^
			Stage 1	0.32^Ab^	1.7^Aa^	tr	0.05^Ac^
*α*-Terpineol	1189	1709	Stage 2	0.07^Aa^	0.93^Aa^	0.04^Ba^	0.11^Aa^
			Stage 3	0.35^Aa^	1.22^Aa^	0.52^Aa^	1.25^Aa^
			Stage 1	0.27^Ab^	0.29^Ab^	0.13^Ac^	0.52^Aa^
cis-Dihydrocarvone	1197	1645	Stage 2	0.28^Aa^	tr	0.1^Aa^	0.02^Ca^
			Stage 3	0.03^Ab^	tr	0^Bc^	0.057^Ba^
			Stage 1	tr	0.04^Ab^	tr	0.07^Aa^
Citronellol	1226	1773	Stage 2	0.07^Aa^	tr	tr	0.01^Ab^
			Stage 3	0.03^ABab^	tr	0.11^Aa^	0.07^Aab^
			Stage 1	0.09^Ab^	0.24^Aa^	tr	tr
Nerol	1228	1797	Stage 2	0.03^Aa^	tr	tr	tr
			Stage 3	0.12^Aa^	tr	tr	0.06^Ab^
			Stage 1	0.07^Ab^	0.78^ABa^	0.79^Aa^	0.0^Ab^
Linalyl acetate	1257	1556	Stage 2	0.09^Ab^	2.61^Aa^	tr	0.09^Ab^
			Stage 3	0.01^Aa^	tr	0.10^Ba^	0.11^Aa^
			Stage 1	tr	tr	tr	0.14^Aa^
Bornyl acetate	1295	1577	Stage 2	tr	tr	1.45^Aa^	tr
			Stage 3	tr	0.01^Aa^	4.21^Aa^	0.07^Ba^
			Stage 1	tr	tr	0.22^Aa^	0.12^Ab^
30 Carvacrol	1302	2239	Stage 2	tr	tr	tr	tr
			Stage 3	0.69^Aa^	0.03^Ab^	0.08^Bb^	0.14^Ab^
			Stage 1	1.4^Aa^	tr	tr	0.02^Ab^
*α*-Terpinyl acetate	1344	1706	Stage 2	0.11^Bab^	0.15^Aab^	tr	0.20^Aa^
			Stage 3	tr	tr	tr	0.72^Aa^
			Stage 1	tr	tr	tr	tr
Geranyl acetate	1383	1765	Stage 2	0.11^Aa^	tr	tr	0.02^Bab^
			Stage 3	0.02^Ac^	0.56^Aa^	0.10^Ac^	0.24^Ab^
			Stage 1	0.047^Bb^	0.04^Ab^	0.19^ABa^	tr
*α*-Humulene	1454	1687	Stage 2	0.03^Bb^	0.02^Ab^	0.16^Ba^	0.01^Bb^
			Stage 3	0.13^Ab^	tr	0.34^Aa^	0.03^Ac^
			Stage 1	0.10^Ba^	0.019^Bc^	0.07^Ab^	tr
Germacrene D	1480	1726	Stage 2	0.25^Aab^	0.89^ABa^	0.12^Ab^	0.03^Bb^
			Stage 3	0.04^Cb^	1.35^Aa^	tr	0.15^Ab^
			Stage 1	0.2^Aa^	tr	tr	tr
Valencene	1495	1740	Stage 2	0.16^Aa^	0.37^Aa^	tr	0.04^ABa^
			Stage 3	0.04^Bb^	0.03^Ab^	tr	0.16^Aa^
			Stage 1	0.21^Ab^	tr	tr	0.17^Ba^
Spathulenol	1577	2121	Stage 2	tr	tr	tr	0.21^Ba^
			Stage 3	tr	tr	tr	2.5^Aa^
			Stage 1	0.21^Ab^	tr	tr	1.18^Aa^
Caryophyllene oxide	1580	2008	Stage 2	0.15^Aa^	tr	tr	tr
			Stage 3	0.08^Aa^	tr	tr	tr
			Stage 1	tr	tr	tr	tr
2Z.6E-Farnesol	1724	2351	Stage 2	tr	tr	tr	0.06^Ba^
			Stage 3	tr	tr	tr	0.79^Aa^
			Stage 1	4.47^Ab^	4.70^Ab^	1.46^Bc^	5.67^Aa^
NI	—	—	Stage 2	5.53^Aa^	1.55^Bc^	2.70^Ab^	1.21^Bc^
			Stage 3	0.45^Bb^	1.85^Ba^	0.37^Cb^	0.12^Bb^

Means of three replicates. (Values with different superscripts are significantly different at *P* < 0.05). Caps superscripts (comparison between stages). Small superscripts (comparison between citrus species).

^A^Components are listed in order of elution in apolar column (HP-5).

RI^a^· RI^b^: retention indices calculated using, respectively, an apolar column (HP-5) and polar column (HPInnowax);

NI: non identified.

**Table 4:** Antibacterial activity of peel essential oils of four cultivars of citrus at different ripening stages against three human pathogenic bacteria.

Bacteria strains	Diameter of inhibition zone including the disc (mm)					
	Ripening stage	Bitter orange	Lemon	Orange maltaise	Mandarin	Gentamicin
*E. coli*	Stage 1	17.30^Ab^	26.60^Aa^	na	26.00^Aa^	27
	Stage 2	na	na	11.30^Ba^	1.00^Cb^	
	Stage 3	14.00^Bab^	17.30^Ba^	17.30^Aa^	16.00^Ba^	
*S. aureus*	Stage 1	16.00^Aa^	3.53^Cb^	na	1.30^Bb^	22
	Stage 2	6.00^Ba^	na	1.36^Bb^	1.06^Bb^	
	Stage 3	14.00^Ac^	16.60^bc^	13.30^Ac^	20.00^Aa^	
*P. aeruginosa*	Stage 1	na	na	na	na	16
	Stage 2	na	na	na	na	
	Stage 3	8.00^b^	13.30^a^	11.30^a^	na	

Means of three replicates. (Values with different superscripts are significantly different at *P* < 0.05). Caps superscripts (comparison between stages). Small superscripts (comparison between citrus species). Na: not active.
